# Timing is everything: the effect of measurement timing on trailing limb angle in gait analysis

**DOI:** 10.3389/fresc.2026.1897235

**Published:** 2026-09-29

**Authors:** Sally Kenworthy, Stacey Gorniak

**Affiliations:** Department of Health and Human Performance, University of Houston, Houston, TX, United States

**Keywords:** biomechanics, clinical assessment, gait, ground reaction force, hemiparesis, propulsion, rehabilitation, stroke

## Abstract

**Introduction:**

Progress towards a meaningful clinical measure of propulsion has been achieved through the trailing limb angle, which describes the position of the trailing limb at the time of peak propulsion. However, variations in the timing of the calculation could limit its clinical translation. The purpose of this study was to compare commonly used approaches to trailing limb angle measurement.

**Methods:**

Nineteen middle-aged adults (mean age [SD]: 56.42 [4.14]) without impairment completed three-dimensional gait analysis while walking on an instrumented split-belt treadmill. Propulsion was defined as the anterior component of the ground reaction force. Peak trailing limb angle was calculated and compared to the angle measured at the time of peak propulsion. Heel strike and toe-off timing were calculated to describe the time of peak propulsion relative to key gait events. Kinematics of the trailing and leading limb hip, knee, and ankle joints were also calculated.

**Results:**

Peak propulsion occurred at 47.50% of the gait cycle, just before contralateral heel strike (49.99%). Peak trailing limb angle (22.77°) was statistically significantly larger than its value when measured at peak propulsion (16.08°, *P* < 0.001), regardless of the distal landmark used for calculation. Peak trailing limb angle occurred just after (.50%) toe off, a statistically significant difference (*P* < 0.05).

**Discussion:**

Our findings challenge the assumption that peak trailing limb angle is equivalent to the trailing limb angle measured at peak propulsion. Calculation methods should reflect this critical distinction to ensure methodological consistency in both research and clinical settings.

## 1 Introduction

Forward movement of the body, known as propulsion, is essential for normal human locomotion and walking speed, enabling efficient movement from point A to point B. When propulsion is impaired, it can significantly reduce walking speed and energy efficiency (1). To understand how propulsion contributes to forward motion, the dynamic model of walking highlights the balance of positive and negative work performed on the body's center of mass (COM) by the leading and trailing limbs (2). During double limb support, the trailing limb generates propulsive forces (positive work) while the leading limb manages the collision forces of weight acceptance (negative work) (3). By overcoming the negative work of the leading limb, the trailing limb ensures smooth and efficient forward progression.

In gait analysis, propulsive forces are often quantified using the anterior component of the ground reaction force (AGRF) (4), which reflects the body's ability to generate sufficient muscular force, range of motion, and positioning to achieve propulsion during walking. Previous studies have shown that AGRF parameters are associated with gait speed (5), severity of hemiparesis (6), and plantar flexor muscle activity (7). Both the impulse and peak magnitude of the AGRF has been used to assess the effectiveness of gait training protocols after stroke as a biofeedback parameter (8–9).

Because most patients with propulsion deficits will be treated outside the research setting, trailing limb angle (10, 11) could be a more accessible point-of-care estimate of propulsion (1). TLA is defined as the angle between the vertical axis of a laboratory and the vector connecting the greater trochanter to the fifth metatarsal head (12). It is a valid estimate of propulsive forces (13), can be measured using a simple set of inertial measurement units (14), is an independent predictor of walking speed (15), and has been effectively used as a biofeedback metric during gait rehabilitation protocols (8). These characteristics make it a viable measure of propulsion as part of a suite of outcomes related to gait function; however, approaches to TLA measurement vary across the literature, particularly in relation to the time at which it is calculated (8, 11, 12, 14–16).

Differences in the timing of TLA calculations in each of these studies (e.g., pre-swing, toe-off, double support, terminal stance) may reflect broad assumptions about the timing of peak propulsion that are not yet supported by the evidence. There is evidence to support the theory that peak propulsion occurs just before contralateral heel strike (HS) (17). The energy cost of human walking decreases when propulsion of the trailing limb occurs just before contralateral HS and increases if it occurs after contralateral HS (17). Generating propulsion at the appropriate time relative to contralateral HS plays a critical role in coordinating the step-to-step transition, and deviations from optimal timing may reflect underlying deficits in motor control quality. Supporting this notion is a study by Malcom et al. which reported optimum energy efficiency when a pneumatic exoskeleton provided propulsive forces prior to contralateral HS rather than after (18). In contrast, a more recent study found that peak AGRF occurred after contralateral HS in young, unimpaired adults, even at varying walking speeds (19).

With conflicting evidence on the timing of peak propulsion and the variability in the timing of TLA calculation methods, it is essential to investigate when propulsion occurs relative to key gait events, such as peak propulsion, contralateral HS, and ipsilateral toe-off (TO). Prior studies characterizing TLA in unimpaired adults have predominantly recruited young adults with mean ages in their mid-twenties (8, 12, 19), limiting the generalizability of their findings to middle-aged populations. The first objective of this study was to describe the timing of peak propulsion in middle-aged, unimpaired adults relative to contralateral HS and ipsilateral TO. The second objective of this study was to determine whether peak TLA occurs at the time of peak AGRF and whether different approaches to TLA calculations result in statistically significant different measurements. Clarifying the timing of peak propulsion and refining TLA measurement methods could enhance gait rehabilitation strategies and improve clinical assessments.

## 2 Methods

A power analysis for this study was completed *a priori* to determine sample size based on a previous study (11). The minimal detectable change (MDC) of AGRF reported was 2.8% body weight and a standard deviation of 4.4% (11). For the current study, the effect size for change in AGRF was set at 0.60 and was calculated using G*Power (Version 3.1.9.4). With the significance criterion of alpha = 0.05 and power = 0.80, the minimum sample size required for this study was 19.

### 2.1 Participants

This study was approved by the University of Houston Institutional Review Board in accordance with the Declaration of Helsinki. Adults between 50 and 65 years of age, who were independent community ambulators and able to consent were recruited to participate in the study. Individuals were excluded if any of the following conditions were present: chronic ankle instability, history of recent (<6 months) ankle sprain, history of orthopedic ankle surgery, chronic foot, ankle, or leg pain, pregnancy, neuropathy, arthritis (osteoarthritis, rheumatoid arthritis, lupus, psoriatic arthritis), inflammation disorders (ankylosing spondylitis, gout), type I or type II diabetes, peripheral vascular disease, stroke, traumatic brain injury, multiple sclerosis, Huntington's disease, post-polio syndrome, Alzheimer's disease or other dementia diagnoses, myasthenia gravis, muscular dystrophy, limb amputation, open ulcer on the lower extremity, Charcot-Marie-Tooth disorder or other hereditary neuropathies, or if the subject requires an assistive device for ambulation. Participants were recruited from the greater Houston area and provided with written informed consent.

### 2.2 Procedures

The data collection session consisted of anthropometric measurements, determination of self-selected walking speed (SSWS) via the 10-meter walk test (10MWT) (20), and three-dimensional (3D) gait analysis. Gait analysis was conducted after a familiarization trial on a split-belt Bertec (Columbus, OH, USA) instrumented treadmill. Participants ambulated using a harness and the treadmill speed was adjusted above or below overground SSWS to accommodate subject comfort prior to data collection trials. A handrail was present during data collection; however, participants were instructed to walk without using the handrail. During data collection, a 16-camera Vicon (Oxford, ENG, UK) motion capture system was used to collect 3D positions of reflective markers of the right and left lower extremities while participants walked at their SSWS for a single three-minute trial. Data collected for analysis was captured from the second minute of the full trial. Ankle, knee, and hip kinematics were collected bilaterally using the Vicon Plug-in Gait Model with the Oxford Foot Model extension. As the Plug-in Gait Model does not include a marker over the greater trochanter (GT), one additional marker captured the proximal landmark used for TLA calculations.

### 2.3 Data processing

Data processing was conducted using custom MATLAB (R2023b, Mathworks Inc., Natick, MA, USA) code. Marker data was captured at 100 Hz and GRF data was captured at 2,000 Hz. All data were time normalized to 100% of the gait cycle (both stance and swing phases) using interpolation. Marker trajectories were smoothed using a Woltring filter in Vicon Nexus software. GRF data was filtered at 10 Hz using a second order Butterworth filter (21). Contralateral heel strike (HS%) and ipsilateral toe-off (TO%) events were identified using a GRF threshold of 30 N (21). All complete gait cycles for the left and right legs within the 1-minute walking trial were included in the analysis.

### 2.4 Dependent variables

Complete definitions of all dependent variables can be found in Table 1. Peak propulsion was defined as the peak amplitude of the AGRF expressed as a percentage of body weight (AGRF_Peak_) for the right and left lower extremities of each participant. TLA was calculated using the two most used definitions: (1) TLA_5M_ = the angle between the vertical axis of the laboratory and a vector connecting the greater trochanter (GT) and the 5th metatarsal head (5M), and (2) TLA_LM_ = the angle between the vertical axis of the laboratory and a vector connecting the GT to the lateral malleolus. Peak amplitude was calculated for AGRF and each TLA measurement technique (AGRF_Peak_, TLA_5MPeak_, and TLA_LMPeak_).

**Table 1:** Variable labels and definitions.

Variable	Definition
AGRF_Peak_	Anterior ground reaction force (AGRF). The peak amplitude of the AGRF normalized to body weight.
AGRF_Peak%_	The percentage of the gait cycle at which AGRF reaches its peak amplitude.
TLA_5M_	Trailing limb angle (TLA). The angle created between the vertical axis of the laboratory and a vector connecting the greater trochanter to the 5th metatarsal head (5M).
TLA_5MPeak_	The peak amplitude of TLA_5M_.
TLA_5MPeak%_	The percentage of the gait cycle at which TLA_5M_ reaches its peak amplitude.
TLA_5MAGRFPeak%_	The amplitude of TLA_5M_ when AGRF reaches its peak amplitude.
TLA_LM_	The angle created between the vertical axis of the laboratory and a vector connecting the greater trochanter to the lateral malleolus (LM).
TLA_LMPeak_	The peak amplitude of TLA_LM_.
TLA_LMPeak%_	The percentage of the gait cycle at which TLA_LM_ reaches its peak amplitude.
TLA_LMAGRFPeak%_	The amplitude of TLA_LM_ when AGRF reaches its peak amplitude.

The timing of the peak value of each variable was expressed as a percentage of the gait cycle (AGRF_Peak%_, TLA_5MPeak%_, and TLA_LMPeak%_). In addition, each TLA measurement was calculated at the time of AGRF_Peak_ (TLA_5MAGRFPeak%_, TLA_LMAGRFPeak%_).

### 2.5 Statistical analysis

SPSS version 29.0 (SPSS IBM, New York, NY, US) was used to conduct statistical analyses. Means and standard deviations (SD) for all dependent variables were calculated for the left and right legs separately for each participant. Prior to pooling the data, the data were assessed for outliers for the left and right legs separately using boxplots and Q-Q plots. The following number of outliers were removed from the data set for the right leg: AGRF_Peak_ (1), AGRF_Peak%_ (1), HS% (1), TO% (1), TLA_LMPeak_ (1), TLA_LMAGRFPeak%_ (2). No outliers were identified for the left leg. Normality of data after outlier removal for each variable was confirmed via a Shapiro–Wilk test (*P* > 0.05). A paired *t*-test was calculated for each variable to determine if there were significant differences between the left and right legs. Significant differences were found between the left and right legs for HS% (*P* = 0.037), TLA_5MPeak_ (*P* = 0.033), TLA_5MPeakAGRF%_ (*P* = 0.031). For comparisons that included any of these three variables, an analysis of variance (ANOVA) was calculated with *Side* (left or right) as a factor with significance level set at *P* < 0.05. For comparisons that utilized the remaining variables without a significant difference between the left and right sides, data from both limbs were pooled prior to analysis. To describe the position of the ankle, knee, and hip joints of the leading and trailing limbs at the time of peak propulsion, means and SD of joint kinematics were calculated at AGRF_Peak%_ and across the entire gait cycle. Effect sizes were calculated using Cohen's *d* for paired *t*-tests and partial eta-squared $\left(\eta_{p}^{2}\right)$ for ANOVA models, with conventional thresholds applied (Cohen's *d*: small = 0.2, medium = 0.5, large = 0.8; $\eta_{p}^{2}$: small = 0.01, medium = 0.06, large = 0.14).

## 3 Results

A total of 19 (11 female, 8 male) middle aged unimpaired adults participated in the study. Most study participants were White (*n* = 13) with the remaining participants reporting Asian (*n* = 4) or Hispanic (*n* = 2) ethnicities. Descriptive statistics of clinical assessment variables are summarized in Table 2.

**Table 2:** Participant clinical assessment variables.

Participant	Age (years)	Height (m)	Mass (kg)	Body mass index	Waist to hip ratio	SSWS (m/s)	SSTS (m/s)	SSTS-SSWS (m/s)	A1C (mg/dL)
1	54	1.71	74	25.17	.74	1.23	1.23	.00	–
2	52	1.56	66	27.05	.93	1.17	1.17	.00	–
3	65	1.77	78	24.85	.90	1.62	1.45	−0.17	5.20
4	59	1.61	63	24.22	.79	1.43	1.28	−0.15	5.50
5	51	1.82	89	26.80	.87	1.36	1.20	−0.16	5.30
6	58	1.79	102	31.81	.99	1.50	1.30	−0.2	5.20
7	55	1.57	50	20.16	.72	1.16	1.16	.00	5.20
8	51	1.55	56	23.33	.80	1.20	1.20	.00	4.50
9	54	1.71	86	29.26	.86	1.39	1.25	−0.14	5.20
10	58	1.82	80	24.26	.80	1.25	1.25	.00	4.90
11	55	1.60	52	20.31	.78	1.29	1.22	−0.07	5.40
12	52	1.80	81	24.91	.92	1.27	1.27	.00	5.50
13	60	1.82	90	27.29	.93	1.62	1.40	−0.22	5.40
14	59	1.81	67	20.46	.82	1.22	1.22	.00	5.50
15	54	1.82	84	25.47	.90	1.33	1.29	−0.04	5.60
16	52	1.67	91	32.38	.82	1.25	1.11	−0.14	5.10
17	62	1.60	49	19.14	.67	0.95	0.95	.00	4.00
18	61	1.59	57	22.62	.77	1.42	1.10	−0.32	5.20
19	60	1.66	61	22.04	.67	0.97	0.97	.00	5.10
Mean	56.42	1.70	72.42	24.82	.83	1.30	1.21	−0.08	5.16
SD	4.14	.10	15.90	3.73	.09	.18	.12	.10	.40

SSWS, self-selected walking speed; SSTS, self-selected treadmill speed; SSTS-SSWS, difference between overground SSWS and SSTS; A1C, hemoglobin A1C test.

Each variable of interest occurred at statistically significantly different times throughout the gait cycle (Tables 3, 4) (Figure 1). There were also statistically significant differences in TLA amplitudes across varying measurement and timing approaches (Tables 3, 4) (Figure 1). There was a significant difference in timing among AGRF_Peak%_ and HS% [*F*_(1,32)_ = 296.260, *P* < 0.001] with a significant effect of side (*F*_(1,32)_ = 5.939, *P* = 0.021). For all other ANOVA tests, there was no effect of *Side*. Joint kinematics of both the trailing and leading limb are presented in Figure 2.

**Table 3:** Results of ANOVA.

Research question	Variable	Mean	SD	Mean difference^a^	*F*	$\eta_{p}^{2}$	*P*
Does AGRF_Peak_ occur before or after contralateral heel strike?	AGRF_Peak%_^b^	47.50%	0.88	−2.49%	296.260	.903	<.001
	HS%^b^	49.99%	0.85				
Does TLA_5M_ peak before or after contralateral heel strike?	TLA_5MPeak%_	57.83%	1.43	7.84%	1,075.614	.971	<.001
	HS%	49.99%	0.85				
Does TLA_LM_ peak before or after contralateral heel strike?	TLA_LMPeak%_	56.07%	1.36	6.08%	727.311	.958	<.001
	HS%	49.99%	0.85				
Is the peak value of TLA_5MPeak_ equivalent to its value at the time of AGRF peak?	TLA_5MPeak_	22.77°	3.03	6.69°	307.650	.988	<.001
	TLA_5MAGRFPeak%_	16.08°	1.77				
Are peak TLA values the same regardless of measurement technique?	TLA_5MPeak_	22.77°	3.03	−5.17°	366.545	.989	<.001
	TLA_LMPeak_	27.94°	2.31				
Are TLA values the same at the time of AGRF peak?	TLA_5MAGRFPeak%_	16.08°	1.77	−7.42°	3,370.145	.989	<.001
	TLA_LMAGRFPeak%_	23.50°	1.88				

a Mean difference = first variable—second variable.

b A significant effect of side was found, see text for full results.

**Table 4:** Results of paired *t*-tests.

Research question	Variable	Mean	SD	Mean difference^a^	*t*	Cohen's *d*	*P*
Does TLA_5M_ peak at the same time as AGRF peak?	AGRF_Peak%_	47.51%	0.87	−10.38%	−42.920	−9.85	<.001
	TLA_5MPeak%_	57.89%	1.44				
Does TLA_LM_ peak at the same time as AGRF peak?	AGRF_Peak%_	47.51%	0.87	−8.61%	−39.968	−9.17	<.001
	TLA_LMPeak%_	56.12%	1.34				
Does AGRF_Peak_ occur before or after ipsilateral TO?	AGRF_Peak%_	47.51%	0.87	−9.88%	−121.924	−27.97	<.001
	TO%	57.39%	1.12				
Does TLA_5M_ peak before or after ipsilateral TO?	TLA_5MPeak%_	57.89%	1.43	.50%	1.909	0.44	<.05
	TO%	57.39%	1.12				
Does TLA_LM_ peak before or after ipsilateral TO?	TLA_LMPeak%_	56.12%	1.34	−1.27%	−5.281	−1.21	<.001
	TO%	57.39%	1.12				
Do both TLA measurement techniques peak at the same time?	TLA_5MPeak%_	57.82%	1.43	1.75%	15.101	3.46	<.001
	TLA_LMPeak%_	56.07%	1.35				
Is the peak value of TLA_LM_ equivalent to its value at the time of AGRF peak?	TLA_LMPeak_	27.88°	2.31	4.20°	15.815	3.63	<.001
	TLA_LMAGRFPeak%_	23.69°	1.75				

a Mean difference = first variable—second variable.

**Figure 1:**
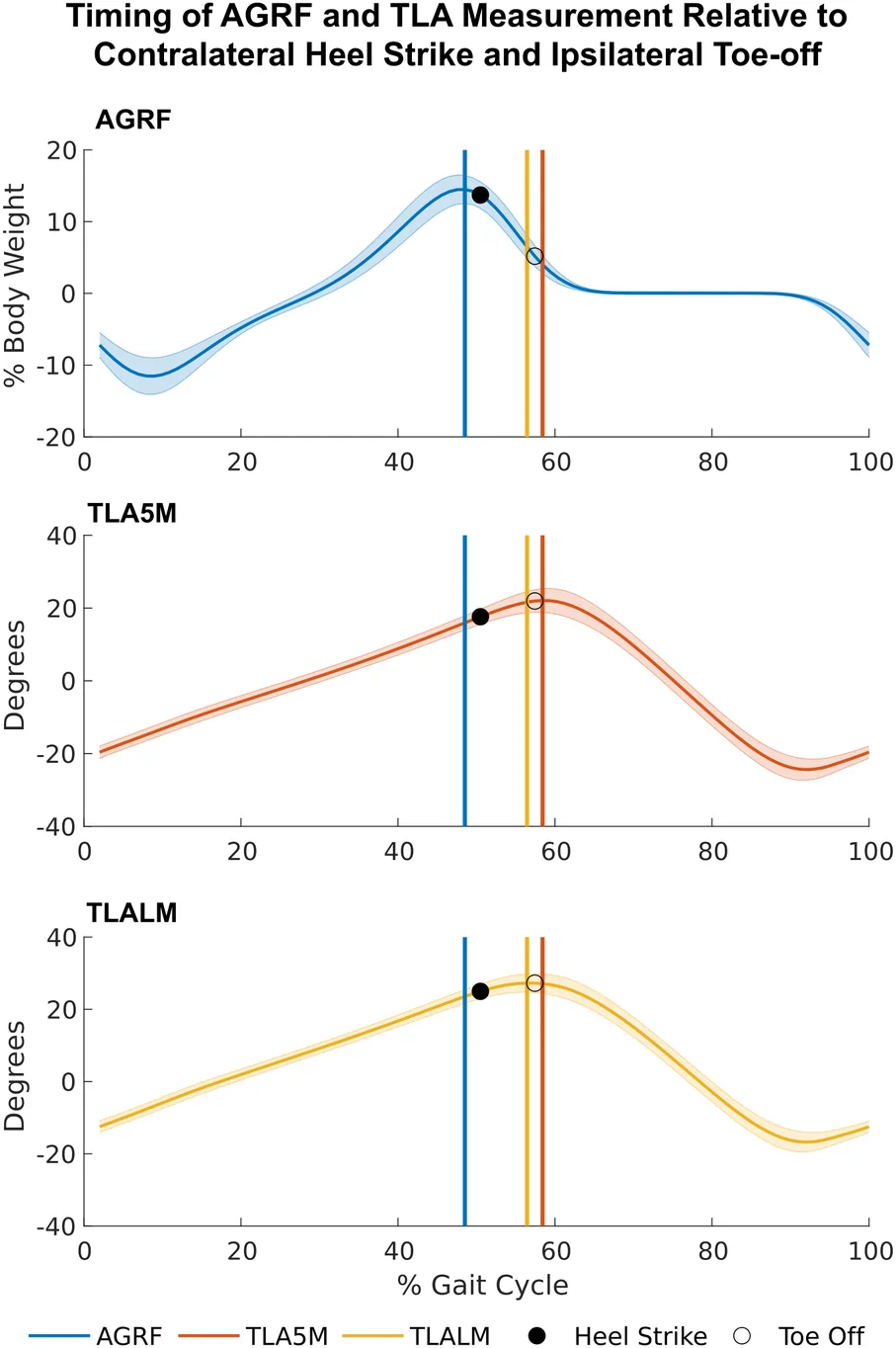
Sample mean (solid lines) and SD (shaded area) of AGRF. Vertical lines represent the timing of the peak of each variable (AGRF, TLA5, TLALM).

**Figure 2:**
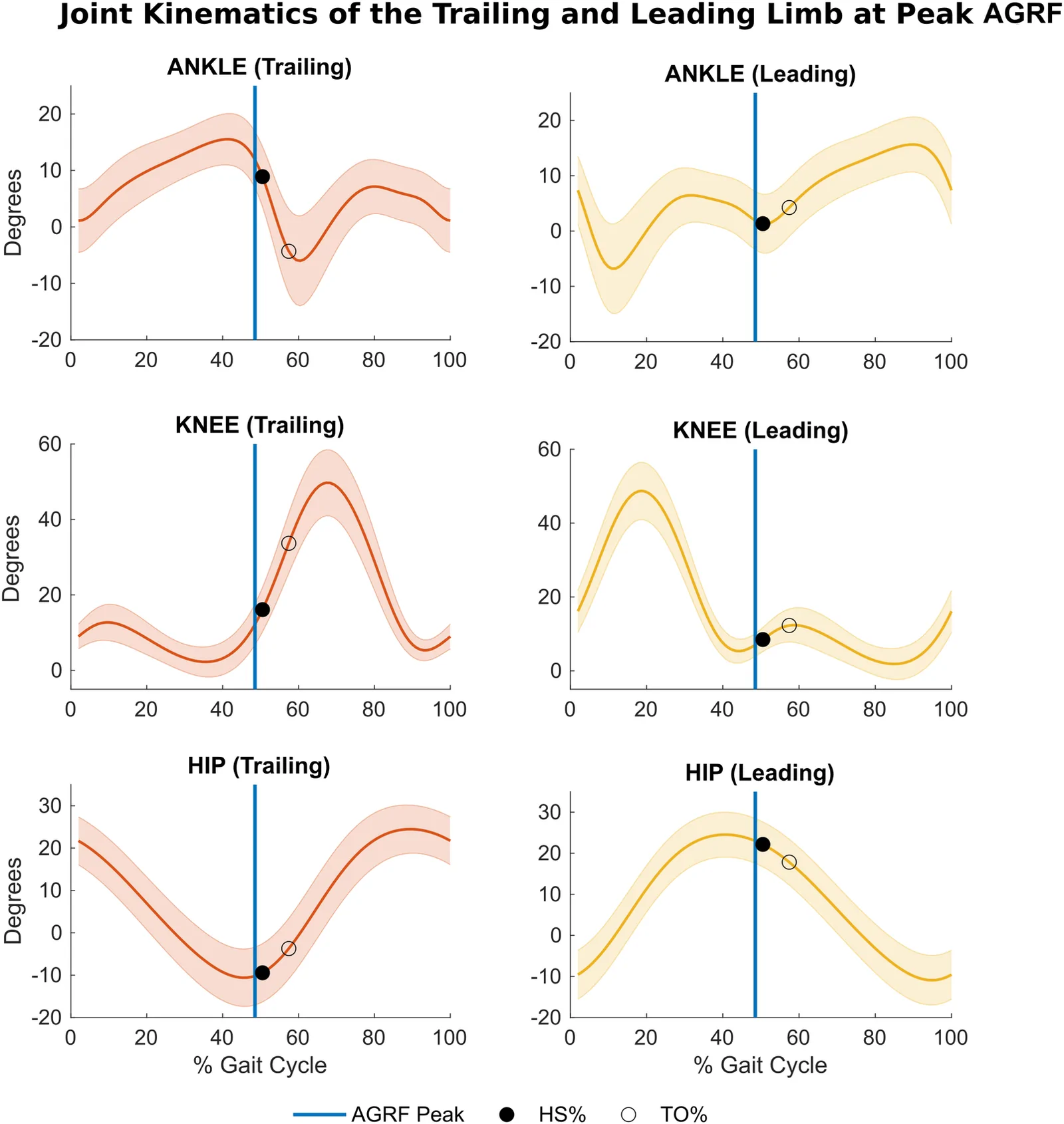
Sample mean (solid lines) and SD (shaded areas) of trailing and leading limb joint kinematics at the time of peak propulsion. Vertical line represents the timing of the AGRF peak. Solid dot = contralateral HS for trailing limb and ipsilateral HS for leading limb. Open dot = ipsilateral TO for trailing limb and contralateral TO for leading limb.

On average, at AGRF_Peak%_, the trailing limb ankle was dorsiflexed (12.07°, ±6.0), the knee was flexed (11.68°, ±4.84), and the hip was extended (−10.24°, ±6.26). At the same time, the leading limb ankle was slightly dorsiflexed (1.81°, ±5.48), the knee was slightly flexed (7.14°, ±3.75), and the hip was flexed (22.18°, ±6.56).

## 4 Discussion

The results of this study showed that peak propulsion occurs just before contralateral HS in a sample of middle-aged unimpaired adults walking at a self-selected speed on a split-belt treadmill. Although the peak value of TLA is often utilized as a measure of peak propulsion (AGRF_Peak_), the current study revealed that peak TLA occurs at a statistically significantly later time in the gait cycle than AGRF_Peak_. The time at which TLA was measured had a significant effect on the resultant angle, regardless of whether the fifth metatarsal head or lateral malleolus was used as the distal landmark of the TLA.

### 4.1 Challenging assumptions around TLA and peak propulsion

Perhaps because previous studies identified TLA as an alternative measure of AGRF_Peak_, there is an assumption that it is the peak value of TLA that represents peak propulsion. A recent state-of-the-art review of 39 studies utilizing TLA as a measure of propulsion found that 70% of the studies provided some timing indicator of measurement, but with substantial variation: toe-off, double support, terminal stance, pre-swing, or peak AGRF (16). The review identified a critical distinction, that none of the 12 studies measuring TLA at “peak TLA” reported measuring it at the time of peak AGRF, yet studies measuring TLA at peak AGRF avoided using the term “peak TLA”. The current study findings validate this important distinction, demonstrating that peak TLA occurs 10.38% later in the gait cycle than TLA measured at peak AGRF.

In the current study, TLA_5M_ at the time of AGRF_Peak_ was on average 16.09° (±1.77°), while the peak value of TLA_5M_ was on average 22.77° (±3.03°), a statistically significant difference. When measured using the lateral malleolus as the distal landmark for TLA, the difference was smaller, but still statistically significant at 4.20°. Additionally, the timing of peak values varied considerably; TLA_5M_ peaked 10.38% later than AGRF_Peak_, and TLA_LM_ peaked 8.61% later. Incorrect timing during instrumented or video gait analysis could lead to a consistent overestimation of TLA, potentially resulting in false negatives. A patient’s propulsive capacity may appear adequate when measured at peak TLA but is actually impaired when measured at the clinical relevant time point of peak propulsion. Our results demonstrate the magnitude of this error: when TLA is measured at peak value rather than at peak AGRF, values are overestimated by an average of 6.69° (using the 5th metatarsal head landmark) or 4.20° (using the lateral malleolus landmark). This misclassification can delay or prevent diagnosis of true propulsive deficits and can obscure whether a gait impairment reflects genuine motor control deficiency or merely compensatory limb positioning. While peak TLA may be adequate for tracking relative progress within a single patient, precision remains critical for establishing normative data, comparing outcomes across facilities, and identifying whether propulsive deficits represent true motor control impairment or a measurement error due to inconsistent timing protocols. Additionally, the proportional relationship between peak TLA and TLA at peak AGRF may be fundamentally altered in individuals with movement disorders, potentially masking clinically meaningful changes in propulsive mechanics if peak TLA is used as a surrogate without verification in impaired populations.

Several studies in this area of research state in their methods sections that TLA was measured at the time of peak AGRF (9, 12, 13, 22–27). However, others reference a specific phase of gait such as toe-off (10, 11, 28–31), double support (15, 32), terminal stance (8, 33); and pre-swing (14). To clarify whether these approaches would create differences in TLA measurement, the current study described AGRF_Peak_ relative to ipsilateral TO, and described the kinematics of the hip, knee, and ankle at the time of AGRF_Peak_. AGRF_Peak_ occurred approximately 2.49% before contralateral HS, and 9.88% before ipsilateral TO, suggesting that peak propulsion does not occur during the period of double support in middle-aged unimpaired adults. The peak value of TLA_5M_ and TLA_LM_ indeed peak very close to ipsilateral TO, with timing differences of only.50% and 1.27%, respectively. Pre-swing is often used interchangeably with TO, and the results show that if TLA is measured at either time, it is capturing the peak value of TLA, rather than TLA at the time of AGRF_Peak_, as originally intended.

Diagrams of the lower limb joint positions in previous studies using TLA are also varied. Some images depict joint positions that are reflective of the double limb support period (10, 14, 24), while others reflect single limb support (23, 32, 34). Our analysis of the trailing and leading limb kinematics in this study lends further insight as to what is functionally happening at the time of peak propulsion in middle-aged unimpaired adults. The results demonstrated that at the time of AGRF_Peak_, the trailing ankle is in a dorsiflexed position, the knee is slightly flexed, and the hip is near a position of maximum extension in the gait cycle. The leading limb ankle is approaching a neutral position for HS, the knee is slightly flexed, and the hip is in a flexed position. These joint positions are reflective of the diagrams that represent the terminal stance phase of gait that occurs during single limb support, just before contralateral HS. In clinical settings where force plates are typically unavailable, clinicians often resort to less rigorous methods like 2D video gait analysis to assess basic temporospatial parameters (35). Future studies should examine the feasibility of the use of wearable sensors to capture the timing of contralateral HS and leading limb joint kinematics as more clinically accessible indicators of peak propulsion.

### 4.2 Gait event detection and peak propulsion considerations

In the current study, the average timing of contralateral HS occurred at approximately 49.99% of the gait cycle while AGRF_Peak_ occurred at approximately 47.50% of the gait cycle. These results are in alignment with the dynamic model of walking that suggests that when push-off occurs before contralateral HS, the energy cost of walking is optimized (2). However, Kuhman et al. found that in a sample of young, unimpaired adults, all measures of propulsion (measured by AGRF_Peak_, peak limb propulsive power, and peak ankle plantar flexion power) occurred after contralateral HS, regardless of walking speed (19). A potential explanation for the conflicting findings is related to data processing decisions, such as HS and TO event identification techniques. In the current study, vertical GRF thresholds (30 N) to identify HS and TO events were chosen in accordance with the study conducted by Revi et al., which validated the use of a minimal set of wearable sensors to provide indirect measurement of the AGRF in both healthy and impaired participants (21). While acceptable HS identification thresholds range from 10 N (36), 20 N (37), to 30 N (21), the choice of the threshold for basic gait events, and decreases in walking speed will impact the timing AGRF_Peak_, and our results should be considered in light of this knowledge. A lower HS threshold (e.g., 10 N) would detect HS earlier, potentially shifting the timing relationships reported in this study. Additionally, individuals with slower walking speed, such as those with stroke, could delay AGRF_Peak_. Therefore, future studies should examine whether the specific timing percentages reported in this study are consistent across unimpaired and impaired populations, and whether these methodological choices systematically change clinical interpretations.

### 4.3 Limitations

Participants in this study had a statistically significantly slower treadmill SSWS (1.2 m/s) than their overground SSWS (1.3 m/s). Although the difference in walking speeds may not be clinically significant, the slower treadmill walking speed suggests that walking on a split belt treadmill in a motion analysis lab could be a challenging task for middle-aged unimpaired adults. It is unknown whether the timing of HS and AGRF_Peak_ would be maintained during overground walking (38). Although self-reported physical activity levels were not measured, 14 of the 19 participants in this study were recruited from a local running club in Houston, Texas. As a result, this sample is not likely representative of the activity levels and overall health of the average adult between 55 and 70 years old, and the results may not be generalizable to middle-aged adults typically at risk of stroke. Notably, some participants showed greater discrepancies between overground SSWS and treadmill SSWS and may be individuals with lower baseline activity levels or general treadmill familiarity. The relatively fast mean SSWS observed in this sample (1.30 m/s) likely reflects the high physical activity levels of many of the participants. As walking speed modulates both TLA magnitude and propulsive force generation (12), the timing relationships described here should be replicated in samples walking at slower speeds. We did, however, capture metabolic health indicators such as waist-to-hip ratio and glycated hemoglobin (A_1c_) levels for future analysis. Significant left-right differences were observed in this study despite participants being unimpaired. These asymmetries likely reflect natural biomechanical variation related to limb dominance or the unfamiliar task of split-belt treadmill walking.

## 5 Conclusion

The findings of this study underscore the critical importance of precise measurement and interpretation of TLA for assessing peak propulsion in gait analysis. This study provides important clarification that whether in the laboratory setting or the clinical setting, the maximum value of TLA is not methodologically equivalent to TLA measurements must be taken at the time of AGRF_Peak,_ which occurs just before contralateral HS in middle-aged unimpaired adults. Our findings provide concrete evidence supporting recommendations for standardized TLA reporting in future research and clinical practice.

## Data Availability

The datasets presented in this study can be found in online repositories. The names of the repository/repositories and accession number(s) can be found below: https://zenodo.org/records/15490514.

## References

[1] Awad LN Lewek MD Kesar TM Franz JR Bowden MG. These legs were made for propulsion: advancing the diagnosis and treatment of post-stroke propulsion deficits. J Neuroeng Rehabil. (2020) 17:139. 10.1186/s12984-020-00747-6 33087137 PMC7579929

[2] Kuo AD Donelan JM. Dynamic principles of gait and their clinical implications. Phys Ther. (2010) 90:157–74. 10.2522/ptj.20090125 20023002 PMC2816028

[3] Donelan JM Kram R Kuo AD. Mechanical work for step-to-step transitions is a major determinant of the metabolic cost of human walking. J Exp Biol. (2002) 205:3717–27. 10.1242/jeb.205.23.3717 12409498

[4] Roelker SA Bowden MG Kautz SA Neptune RR. Paretic propulsion as a measure of walking performance and functional motor recovery post-stroke: a review. Gait Posture. (2019) 68:6–14. 10.1016/j.gaitpost.2018.10.027 30408710 PMC6657344

[5] Bowden MG Balasubramanian CK Behrman AL Kautz SA. Validation of a speed-based classification system using quantitative measures of walking performance poststroke. Neurorehabil Neural Repair. (2008) 22:672–5. 10.1177/1545968308318837 18971382 PMC2587153

[6] Bowden MG Balasubramanian CK Neptune RR Kautz SA. Anterior-posterior ground reaction forces as a measure of paretic leg contribution in hemiparetic walking. Stroke. (2006) 37:872–6. 10.1161/01.STR.0000204063.75779.8d 16456121

[7] Turns LJ Neptune RR Kautz SA. Relationships between muscle activity and anteroposterior ground reaction forces in hemiparetic walking. Arch Phys Med Rehabil. (2007) 88:1127–35. 10.1016/j.apmr.2007.05.027 17826457 PMC2367107

[8] Liu J Santucci V Eicholtz S Kesar TM. Comparison of the effects of real-time propulsive force versus limb angle gait biofeedback on gait biomechanics. Gait Posture. (2021) 83:107–13. 10.1016/j.gaitpost.2020.10.014 33129170 PMC7787119

[9] Ray NT Knarr BA Higginson JS. Walking speed changes in response to novel user-driven treadmill control. J Biomech. (2018) 78:143–9. 10.1016/j.jbiomech.2018.07.035 30078637 PMC6129197

[10] Tyrell CM Roos MA Rudolph KS Reisman DS. Influence of systematic increases in treadmill walking speed on gait kinematics after stroke. Phys Ther. (2011) 91:392–403. 10.2522/ptj.20090425 21252308 PMC3048817

[11] Kesar TM Binder-Macleod SA Hicks GE Reisman DS. Minimal detectable change for gait variables collected during treadmill walking in individuals post-stroke. Gait Posture. (2011) 33:314–7. 10.1016/j.gaitpost.2010.11.024 21183350 PMC3042506

[12] Hsiao H Knarr BA Higginson JS Binder-Macleod SA. The relative contribution of ankle moment and trailing limb angle to propulsive force during gait. Hum Mov Sci. (2015) 39:212–21. 10.1016/j.humov.2014.11.008 25498289 PMC4272868

[13] Lewek MD Sawicki GS. Trailing limb angle is a surrogate for propulsive limb forces during walking post-stroke. Clin Biomech. (2019) 67:115–8. 10.1016/j.clinbiomech.2019.05.011 PMC6635006 31102839

[14] Miyazaki T Kawada M Nakai Y Kiyama R Yone K. Validity of measurement for trailing limb angle and propulsion force during gait using a magnetic inertial measurement unit. Biomed Res Int. (2019) 2019:8123467–8. 10.1155/2019/8123467 31930138 PMC6942796

[15] Awad LN Binder-Macleod SA Pohlig RT Reisman DS. Paretic propulsion and trailing limb angle are key determinants of long-distance walking function after stroke. Neurorehabil Neural Repair. (2015) 29:499–508. 10.1177/1545968314554625 25385764 PMC4426250

[16] Kenworthy SA Gorniak SL. Trailing limb angle as a clinically feasible measure of propulsion: a state-of-the-art review. Gait Posture. (2026) 123:110002. 10.1016/j.gaitpost.2025.110002 41066975

[17] Kuo AD. Energetics of actively powered locomotion using the simplest walking model. J Biomech Eng. (2002) 124:113–20. 10.1115/1.1427703 11871597

[18] Malcolm P Derave W Galle S De Clercq D. A simple exoskeleton that assists plantarflexion can reduce the metabolic cost of human walking. PLoS One. (2013) 8:e56137. 10.1371/journal.pone.0056137 23418524 PMC3571952

[19] Kuhman D Hurt CP. The timing of locomotor propulsion in healthy adults walking at multiple speeds. Hum Mov Sci. (2019) 68:102524. 10.1016/j.humov.2019.102524 31733429

[20] Peters DM Fritz SL Krotish DE. Assessing the reliability and validity of a shorter walk test compared with the 10-meter walk test for measurements of gait speed in healthy, older adults. J Geriatr Phys Ther. (2013) 36:24–30. 10.1519/JPT.0b013e318248e20d 22415358

[21] Revi DA Alvarez AM Walsh CJ De Rossi SMM Awad LN. Indirect measurement of anterior-posterior ground reaction forces using a minimal set of wearable inertial sensors: from healthy to hemiparetic walking. J Neuroeng Rehabil. (2020) 17:82. 10.1186/s12984-020-00700-7 32600348 PMC7322880

[22] Alingh JF Groen BE Kamphuis JF Geurts ACH Weerdesteyn V. Task-specific training for improving propulsion symmetry and gait speed in people in the chronic phase after stroke: a proof-of-concept study. J Neuroeng Rehabil. (2021) 18:69. 10.1186/s12984-021-00858-8 33892754 PMC8062933

[23] Farrens AJ Marbaker R Lilley M Sergi F. Training propulsion: locomotor adaptation to accelerations of the trailing limb. IEEE Int Conf Rehabil Robot. (2019) 2019:59–64. 10.1109/ICORR.2019.8779374 31374607

[24] Hedrick EA Parker SM Hsiao H Knarr BA. Mechanisms used to increase propulsive forces on a treadmill in older adults. J Biomech. (2021) 115:110139. 10.1016/j.jbiomech.2020.110139 33321429 PMC7855876

[25] Hsiao H Knarr BA Higginson JS Binder-Macleod SA. Mechanisms to increase propulsive force for individuals poststroke. J Neuroeng Rehabil. (2015) 12:40. 10.1186/s12984-015-0030-8 25898145 PMC4406180

[26] Lewek MD Raiti C Doty A. The presence of a paretic propulsion reserve during gait in individuals following stroke. Neurorehabil Neural Repair. (2018) 32:1011–9. 10.1177/1545968318809920 30558525 PMC6300055

[27] McGrath RL Ziegler ML Pires-Fernandes M Knarr BA Higginson JS Sergi F. The effect of stride length on lower extremity joint kinetics at various gait speeds. PLoS One. (2019) 14:e0200862. 10.1371/journal.pone.0200862 30794565 PMC6386374

[28] Browne MG Stenum J Padmanabhan P Roemmich RT. Simple within-stride changes in treadmill speed can drive selective changes in human gait symmetry. PLoS One. (2023) 18:e0287568. 10.1371/journal.pone.0287568 37883477 PMC10602355

[29] Moradian N Ko M Hurt CP Brown DA. Effects of backward-directed resistance on propulsive force generation during split-belt treadmill walking in non-impaired individuals. Front Hum Neurosci. (2023) 17:1214967. 10.3389/fnhum.2023.1214967 38111676 PMC10725924

[30] Rendos NK Zajac-Cox L Thomas R Sato S Eicholtz S Kesar TM. Verbal feedback enhances motor learning during post-stroke gait retraining. Top Stroke Rehabil. (2021) 28:362–77. 10.1080/10749357.2020.1818480 32942960 PMC7969478

[31] Tsushima Y Fujita K Miaki H Kobayashi Y. Effects of increasing non-paretic step length on paretic leg movement during hemiparetic gait: a pilot study. J Phys Ther Sci. (2022) 34:590–5. 10.1589/jpts.34.590 35937629 PMC9345751

[32] McCain EM Dick TJM Giest TN Nuckols RW Lewek MD Saul KR. Mechanics and energetics of post-stroke walking aided by a powered ankle exoskeleton with speed-adaptive myoelectric control. J Neuroeng Rehabil. (2019) 16:57. 10.1186/s12984-019-0523-y 31092269 PMC6521500

[33] Mitsutake T Nakazono H Yoshizuka H Taniguchi T Sakamoto M. Increased trailing limb angle is associated with regular and stable trunk movements in patients with hemiplegia. J Stroke Cerebrovasc Dis. (2022) 31:106242. 10.1016/j.jstrokecerebrovasdis.2021.106242 34915305

[34] Balasubramanian CK Neptune RR Kautz SA. Foot placement in a body reference frame during walking and its relationship to hemiparetic walking performance. Clin Biomech. (2010) 25:483–90. 10.1016/j.clinbiomech.2010.02.003 PMC2881577 20193972

[35] Michelini A Eshraghi A Andrysek J. Two-dimensional video gait analysis: a systematic review of reliability, validity, and best practice considerations. Prosthet Orthot Int. (2020) 44:245–62. 10.1177/0309364620921290 32507049

[36] Tirosh O Sparrow WA. Identifying heel contact and toe-off using forceplate thresholds with a range of digital-filter cutoff frequencies. J Appl Biomech. (2003) 19:178–84. 10.1123/jab.19.2.178

[37] Zeni JA Jr Richards JG Higginson JS. Two simple methods for determining gait events during treadmill and overground walking using kinematic data. Gait Posture. (2008) 27:710–4. 10.1016/j.gaitpost.2007.07.007 17723303 PMC2384115

[38] Lee SJ Hidler J. Biomechanics of overground vs. treadmill walking in healthy individuals. J Appl Physiol. (2008) 104:747–55. 10.1152/japplphysiol.01380.2006 18048582

